# The gender gap in fair earnings increases with age due to higher age premium for men

**DOI:** 10.1111/1468-4446.13149

**Published:** 2024-09-18

**Authors:** Jule Adriaans, Carsten Sauer, Katharina Wrohlich

**Affiliations:** ^1^ Faculty of Sociology Bielefeld University Bielefeld Germany; ^2^ Research Group Gender Economics German Institute for Economic Research (DIW Berlin) University of Potsdam Potsdam Germany

**Keywords:** distributive justice, factorial survey experiment, gender bias, gender inequality

## Abstract

This study explores how gender and age interact in shaping beliefs about fair pay through a factorial survey experiment conducted with German employees. Respondents evaluated hypothetical worker descriptions varying in age, gender, and earnings. While no gender gap in fair earnings was found for the youngest hypothetical workers, a significant gap favoring men emerged with increasing age. This suggests that male workers receive a higher age premium on fair earnings than female workers. The findings highlight the need to understand how gender interacts with other characteristics to legitimize workplace inequalities.

## 1 INTRODUCTION

Gender inequalities in the workplace are well‐documented, with the persistent gender pay gap being a prominent example. Social scientists have long tried to understand the roots and mechanisms behind this gap. Former studies have explored individual characteristics like education and tenure, labor market segregation, or direct discrimination. However, there is growing interest in examining how beliefs about legitimate differences in earnings contribute to and perpetuate this gap (Blau & Kahn, 2017; Ridgeway, 2011).

Recent empirical studies have taken a new approach by investigating these beliefs through fairness evaluations of others' pay. Factorial survey experiments present respondents with descriptions of hypothetical workers who receive hypothetical earnings. This method allows researchers to measure which worker characteristics are perceived as crucial for fair pay assessments. Justice theory (Liebig & Sauer, 2016) suggests that respondents rely on various factors in forming these evaluations, such as meritocracy norms—where higher contributions warrant higher pay—and diffuse status characteristics like gender, where being male is often associated with deserving higher pay (Adriaans et al., 2023; Auspurg et al., 2017).

Several factorial survey studies have shown that respondents produce a gap in their fairness judgments favoring men (Auspurg et al., 2017; Gatskova, 2015; Jann, 2008; Lang & Groß, 2020; Sauer, 2020). This gap is particularly pronounced when the hypothetical workers are described as married (Jann et al., 2021), indicating that gender may interact with other personal characteristics in shaping fair compensation beliefs.

This paper focuses on the interaction between gender and age in shaping beliefs about fair pay. This focus is motivated by two observations: First, age serves as a status marker (Walker, 2022) and a proxy for work experience, which is important for fairness assessments (Auspurg et al., 2017). Second, research shows that gender pay gaps increase with age. In Germany, gender pay gaps increase sharply after the age of 30, partly due to the unequal distribution of paid and unpaid care work (Cukrowska‐Torzewska & Matysiak, 2020; Schäper et al., 2023). Women often have prolonged career interruptions following childbirth and are more likely to work part‐time. In Germany, wage returns to part‐time work experience are much lower compared to full‐time work experience (Blesch et al., 2023) and similar patterns are documented for other high‐income countries such as the UK (Blundell et al., 2016).

If indeed longer and more pronounced career interruptions are presumed for women, reducing their perceived experience and seniority, this should result in growing gender differences in fair earnings over the life‐course. On the other hand, a woman's age may also be used as a proxy for the likelihood of the presence of young children in the household and correspondingly higher levels of care work. If this is the case, gender differences in perceived fair earnings should diminish for older workers.

Building on the joint relevance of age and gender in determining wages, we use a factorial survey experiment implemented in a large‐scale employee survey conducted in Germany to explore: are there gender‐specific age premiums on perceived fair earnings?

## 2 DATA AND ANALYTICAL STRATEGY

### 2.1 Data

Our data stem from the employee panel study “Legitimation of Inequality over the Life‐span” (LINOS), which was first conducted in the winter of 2012/2013 and repeated in 2017. The LINOS study surveyed a representative sample of employees in Germany who were subject to social security contributions as of December 31, 2011. In the first wave, the survey institute interviewed 4731 respondents (Sauer & Valet, 2014). 3607 respondents consented to be invited for a second wave. Of these respondents, 2741 were successfully re‐interviewed in 2017 (Adriaans et al., 2019).

To explore our research question, we rely on a factorial survey experiment on the fairness of earnings (Liebig et al., 2015) that we implemented in a randomly selected 50% subsample of wave 1 respondents (*N* = 2253) who fulfilled the same task again in wave 2 (*N* = 1329). In the factorial survey experiment, we asked respondents to evaluate the fairness of earnings received by fictitious employees. Each of these employee descriptions—called vignettes—experimentally varied seven dimensions: age, gender, occupation, type of staff, job performance, unemployment level in the occupation, and monthly gross earnings (see Figure A1 for an example vignette and Table A1 for an overview of dimensions and levels in online Supporting Information S1: Appendix A). Following recommendations on the optimal design of vignettes (Treischl & Wolbring, 2022), the seven vignette dimensions were chosen to provide plausible worker descriptions combining worker characteristics that have been document as relevant for justice judgments in past research (e.g., Auspurg et al., 2017; Sauer, 2020).

Out of all potential combinations of levels, we sampled 100 vignettes via a D‐efficient algorithm. We grouped these 100 vignettes into 10 decks with 10 vignettes each. Each respondent evaluated one deck of vignettes presented in random order. The respondent's task was to rate the fairness of earnings of each fictitious employee using an 11‐point response scale that ranged from −5 “unjustly low” to +5 “unjustly high,” with the scale mid‐point (0 “just”) indicating perfect fairness. Item nonresponse for the vignette evaluations was 3.4%.

We pool data from both waves of the LINOS study. After listwise deletion of missing values, the analysis sample consists of 35,585 vignette evaluations from 2284 respondents. All analyses relied on the scientific use files of the LINOS study (Liebig et al., 2014, 2019) and readers can find the complete replication code at https://osf.io/xymve/.

The experimental design ensured that all vignette characteristics were uncorrelated (see Table A2 in online Supporting Information S1: Appendix A). Thus, the design allows us to estimate the effect of each vignette dimension on the fairness evaluation of earnings that respondents provided.

### 2.2 Analytical strategy

Our research question focuses on the effect of the vignette dimensions of *age* and *gender* on the fairness of earnings. The fairness evaluation thus serves as the outcome variable, with vignette persons' *gender* and *age* as explanatory variables. Fictitious vignette persons were male (0) or female (1). The vignette dimension *age* had three levels: 30 years, 45 years, and 60 years, corresponding to different career‐ and life stages. We control for all other vignette person characteristics to isolate the effect of *age* and *gender*, including their *occupation* ^1^ (we coded the levels into values on the Standard International Occupational Prestige Scale (SIOPS)), whether they belonged to “core *staff*” or “temporary *staff*,” their individual *job performance* (levels: “below‐average performance,” “average performance,” or “above‐average performance”), the *unemployment rate* in their occupation (levels: “low” or “high”), and their *earnings* (included as natural logs of monthly gross earnings).

Our analysis proceeds in three steps. First, we regressed the fairness evaluation of earnings on all vignette dimensions to capture the main effect of vignette persons' *gender*. In a second step, we included the interaction between vignette dimensions of *age* and *gender* to test for gender‐specific returns to *age*. Finally, we ran separate models for male and female respondents to test for heterogenous effects. All models included a dummy variable indicating the survey wave and clustered standard errors at the individual level, considering that respondents evaluated multiple vignettes.

We report predictive margins that are transformed into earnings metrics for better readability and illustration of effect sizes.^2^ Online Supporting Information S1: Appendix B includes the full regression tables.

## 3 RESULTS

**FIGURE 1:**
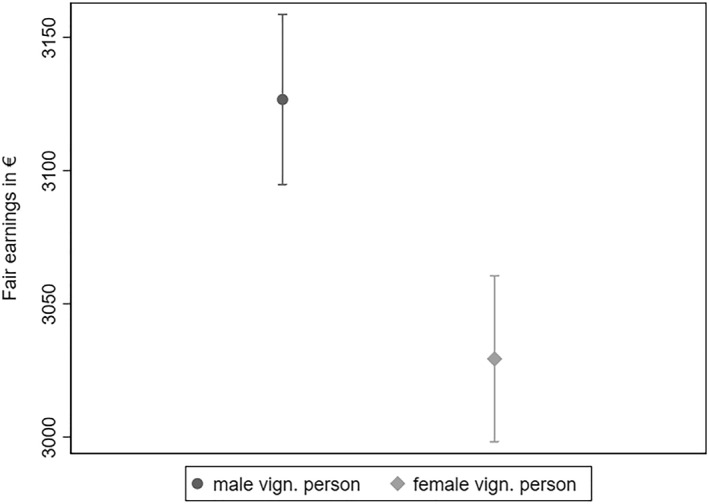
Fair earnings for male and female vignette persons. Data: LINOS‐1 & LINO‐2 (pooled). Fairness evaluation of earnings regressed on vignette dimensions (Model 1 in Table B1 of Appendix B). 95% confidence intervals.

Figure 1 illustrates the fair gender pay gap documented in previous research (Adriaans et al., 2020; Auspurg et al., 2017; Sauer, 2020). Keeping all characteristics of the vignette constant, the fair earnings for male vignette persons were 3127€. In contrast, the fair earnings for female vignette persons stood at 3029€, translating into a fair gender pay gap of about 3%.^3^

**FIGURE 2:**
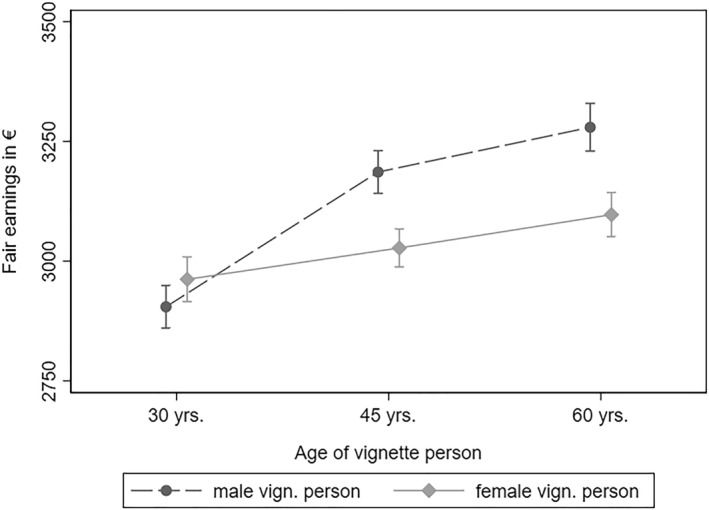
Fair earnings for male and female vignette persons by age. Data: LINOS‐1 & LINO‐2 (pooled). Fairness evaluation of earnings regressed on vignette dimensions (Model 2 in Table B1 of Appendix B). 95% confidence intervals.

Figure 2 illustrates the interaction effect between the vignette person's *age* and *gender*. For the youngest vignette persons (30 years old), fair earnings did not differ significantly by gender. From this, we can conclude that respondents did not view it as fair when young male vignette persons received higher earnings than young female vignette persons. However, respondents did produce gender gaps in fair earnings when evaluating vignette persons who were 45 or 60 years old. While 30‐year‐old vignette persons seem to start with the same fair earnings irrespective of their gender, respondents allocate a higher age premium to male vignette persons: Fair earnings for 60‐year‐old male vignette persons were about 375€ or 11.4% higher compared to 30‐year‐old male vignette persons, keeping all other characteristics of the vignette person constant. At the same time, fair earnings for female vignette persons who were 60 years old were only about 4.4% or 135€ higher compared to 30‐year‐old female vignette persons. Gendered age premiums thus seem essential in explaining the overall gender gap in fair earnings. Online Supporting Information S1: Appendix C illustrates that this pattern is particularly pronounced for older respondents but that the general trend is consistent for respondents of different ages.

**FIGURE 3:**
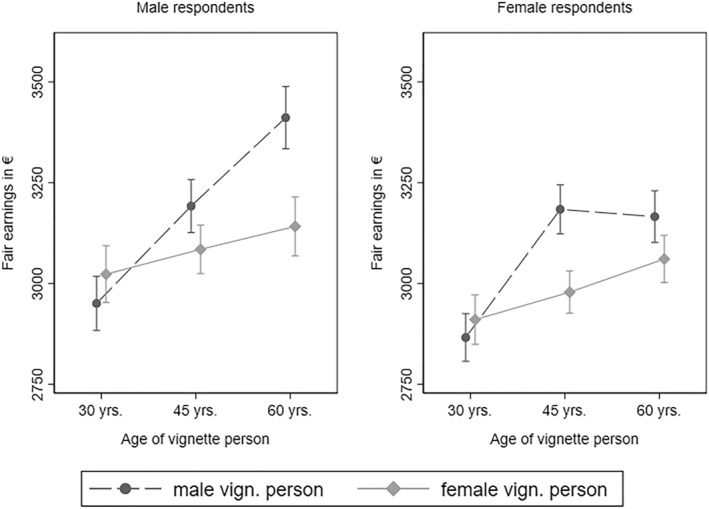
Fair earnings for male and female vignette persons, by age of vignette person and gender of respondents. Data: LINOS‐1 & LINO‐2 (pooled). Fairness evaluation of earnings regressed on vignette dimensions (Model 3 and Model 4 in Table B1 of Appendix B). 95% confidence intervals.

To test for potential heterogeneous effects by respondent gender, we ran separate models for male and female respondents (see Figure 3). Similar to findings reported in Figure 2, neither male nor female respondents produced a gender gap in fair earnings for the youngest vignette persons. However, male and female respondents assigned comparatively higher age premiums to male vignette persons, eventually producing gender gaps in fair earnings. While gender gaps in fair earnings are largest for 60‐year‐old vignette persons among male respondents, female respondents produce the largest gender gap in fair earnings for 45‐year‐old vignette persons.

Age premiums for female vignette persons generally follow a similar trend among male and female respondents. For female respondents, the difference in fair wages for 60‐year female vignette persons compared to 30‐year‐olds was about 4.9%. For male respondents, this difference was about 3.8%. More notable, however, were gender differences in age premiums assigned to male vignette persons. For male respondents, the difference in fair wages for 60‐year male vignette persons compared to 30‐year‐olds was about 13.5%, following an almost linear increase in fair earnings over vignette persons' age. Among female respondents, however, after an age premium of almost 10% for 45‐year‐old male vignette persons compared to their 30‐year‐old counterparts, there is no additional age premium for 60‐year‐old male vignette persons.

## 4 CONCLUSION

Our findings reveal age‐related dynamics in gendered perceptions of fair earnings. While young men and women are perceived to deserve similar earnings, this parity diminishes with age. Men receive increasing age premiums on fair earnings, reflecting societal expectations of seniority and experience, whereas women's fair earnings remain relatively stable across different age groups.

This smaller return for age in life‐course fair earnings for female workers is observed among both male and female respondents. One possible explanation for these findings is that respondents anticipate career interruptions for women due to family or care responsibilities, leading to an assumption of lower experience and seniority for older female workers. This mirrors real‐world patterns where women face pronounced career breaks and part‐time work in mid‐age, contributing to a widening pay gap as they age (Schäper et al., 2023).

Conversely, the linear increase in male vignette persons' fair earnings observed for male respondents suggests an expectation of continuous professional growth and increasing rewards for men. The non‐linear pattern observed among female respondents, with a peak in fair earnings for middle‐aged men, might reflect a societal role expectation of men as family providers. This interpretation aligns with other studies' findings that married men, especially married men with children, receive higher fair earnings (Jann et al., 2021; Lang & Groß, 2020).

These findings have significant implications for sociologists studying gender inequality. They highlight the need for a nuanced understanding of how gendered ideas of fairness differ over the life course to comprehend their contribution to persistent gender disparities in the workplace. Future research using longitudinal studies and cross‐cultural comparisons could provide further insights into how these dynamics evolve and differ across contexts. By unpacking how gender intersects with other worker characteristics in shaping fair earnings perceptions, we can better understand the implicit assumptions and biases that legitimize inequalities at work and inform policies that promote gender equality.

## CONFLICT OF INTEREST STATEMENT

We declare that there are no conflicts of interest.

## Supporting information

Supporting Information S1

## Data Availability

All analyses rely on factually anonymized scientific use files of the LINOS study (10.25652/diw_data_S0026.1; 10.25652/diw_data_S0027.1). Data access is available through the Research Data Center FDZ‐BO (https://portal.fdz‐bo.diw.de) at the German Institute for Economic Research (DIW Berlin). Full replication code is available here: https://osf.io/xymve/.

## ENDNOTES

^1^
The occupations presented to respondents were chosen to be easily recognizable by respondents as varying in prestige. In order to present respondents with plausible and realistic scenarios, we excluded occupations that are dominated by public sector employment or collective wage agreements where large variations in pay are rare. These requirements resulted in a list of occupations that included both gender‐mixed (insurance clerk and unskilled worker) and male‐dominated occupations (auto mechanic, programmer, and production manager) that also varied in their gender pay ratios.

^2^
We transformed the regression coefficients into the earnings metric with exp(b_*x*_/−b_earnings(ln)_). For more details on this transformation, see Auspurg et al. (2017), as well as the replication code provided along with the manuscript.

^3^
Based on data from general population surveys conducted in Germany in 2008 and 2009, both Auspurg et al. (2017) as well as Lang and Groß (2020) have documented fair gender pay gaps of about 8 percent, while no fair gender pay gap was found in a large student sample with data collected in 2008 (Sauer, 2020). In contrast to these studies, the LINOS study surveyed only employed respondents who are—on average—older than respondents in the student sample and younger than respondents in general population samples that also include a large share of retirees. This may explain the smaller gender bias observed in the LINOS data compared to general population studies. Figure C1 in the online Appendix C further shows that fair gender pay gaps are larger among older respondents in the LINOS study.
