# Supplementary material for: The gender gap in fair earnings increases with age due to higher age premium for men
Source: Br J Sociol. 2024 Sep 18;76(1):180–7. doi: 10.1111/1468-4446.13149 (PMC11717164; doi:10.1111/1468-4446.13149)
Supplement: Supplementary file 1 — Supporting Information S1 [file BJOS-76-180-s001.docx]

Online appendix to the research note “The gender gap in fair earnings increases with age due to higher age premium for men”

# Online Appendix A


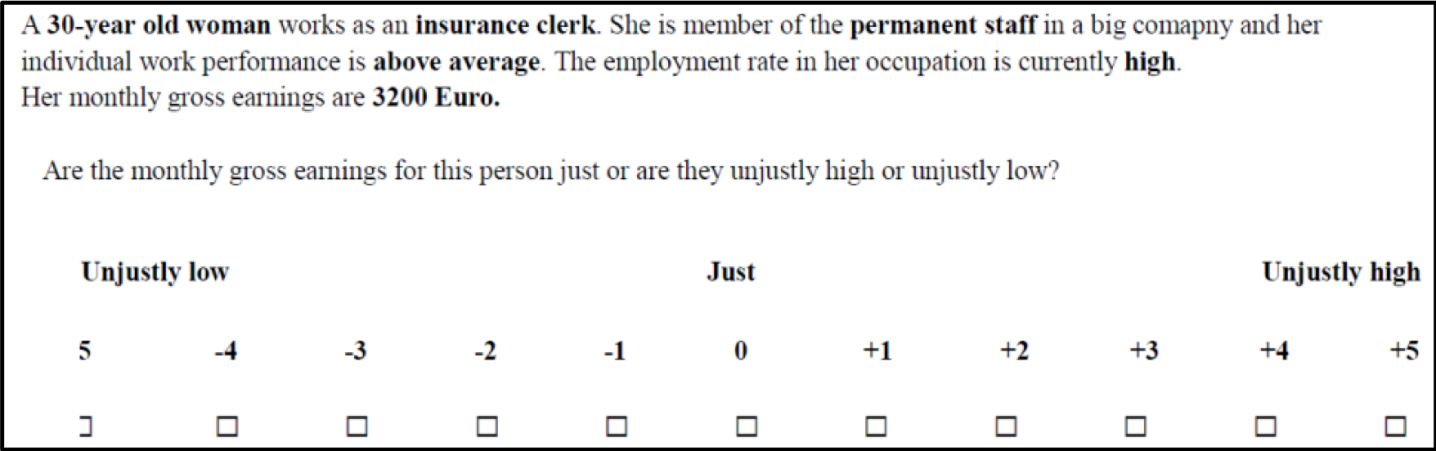


**Figure A1.** Example vignette.

**Table A1.** Dimensions and levels of the vignettes.

| # | Dimensions | Levels |
| --- | --- | --- |
| 1 | Age | 30/45/60 years |
| 2 | Gender | Female/male |
| 3 | Occupation^a^ | Unskilled worker (18)/auto mechanic (43)/insurance clerk (44)/ programmer (51)/production manager (60) |
| 4 | Staff membership | permanent/temporary staff |
| 5 | Individual job performance | Below average/average/above average |
| 6 | Unemployment rate in occ. | Low/high |
| 7 | Gross monthly earnings | 1500/2400/3200/4600/6500 Euro |

Note: ^a^ Corresponding values on the Standard International Occupational Prestige Scale (SIOPS) are presented in brackets.

**Table A2.** Pairwise correlation of vignette dimensions.

| Vignette dimensions | (1) | (2) | (3) | (4) | (5) | (6) | (7) |
| --- | --- | --- | --- | --- | --- | --- | --- |
| (1) Age | 1.00 |  |  |  |  |  |  |
| (2) Gender | 0.00 | 1.00 |  |  |  |  |  |
| (3) Occupation (SIOPS) | -0.01 | -0.01 | 1.00 |  |  |  |  |
| (4) Staff membership | -0.01 | -0.01 | 0.03 | 1.00 |  |  |  |
| (5) Ind. Job performance | -0.01 | 0.00 | 0.03 | 0.01 | 1.00 |  |  |
| (6) Unemployment rate in occ. | -0.03 | 0.01 | -0.02 | 0.01 | 0.03 | 1.00 |  |
| (7) Gross monthly earnings (log) | 0.00 | 0.01 | 0.00 | -0.01 | 0.00 | -0.01 | 1.00 |

# Online Appendix B

**Table B1.** Fairness evaluation regressed on vignette dimensions.

|  | (1) | | (2) | | (3) | | (4) | |
| --- | --- | --- | --- | --- | --- | --- | --- | --- |
|  | Main effects model  Full Sample | | Gender x Age  Full Sample | | Gender x Age  Male respondents | | Gender x Age  Female respondents | |
|  | b | se | b | se | b | se | b | se |
| *Vignette Dimensions* |  |  |  |  |  |  |  |  |
| Age: 30 years | Ref. |  | Ref. |  | Ref. |  | Ref. |  |
| Age: 45 years | -0.219^***^ | (0.023) | -0.354^***^ | (0.036) | -0.298^***^ | (0.053) | -0.408^***^ | (0.050) |
| Age: 60 years | -0.317^***^ | (0.025) | -0.465^***^ | (0.038) | -0.549^***^ | (0.056) | -0.386^***^ | (0.052) |
| Gender: Female | 0.121^***^ | (0.019) | -0.075 | (0.040) | -0.0919 | (0.061) | -0.0597 | (0.053) |
| Occupation (SIOPS)^a^ | -0.426^***^ | (0.009) | -0.426^***^ | (0.009) | -0.459^***^ | (0.013) | -0.395^***^ | (0.012) |
| Staff (1=temporary) | 0.237^***^ | (0.021) | 0.256^***^ | (0.022) | 0.255^***^ | (0.032) | 0.256^***^ | (0.029) |
| Performance: below avg. | 0.818^***^ | (0.025) | 0.819^***^ | (0.025) | 0.783^***^ | (0.037) | 0.854^***^ | (0.034) |
| Performance: avg. | Ref. |  | Ref. |  | Ref. |  | Ref. |  |
| Performance: above avg. | -0.779^***^ | (0.023) | -0.786^***^ | (0.024) | -0.735^***^ | (0.034) | -0.832^***^ | (0.033) |
| Unemployment rate: low | 0.00151 | (0.017) | 0.00175 | (0.017) | 0.00325 | (0.026) | 0.00262 | (0.023) |
| Gross earnings (log) | 3.835^***^ | (0.030) | 3.833^***^ | (0.030) | 3.783^***^ | (0.045) | 3.877^***^ | (0.041) |
| Survey year: 2017 | -0.234^***^ | (0.026) | -0.234^***^ | (0.026) | -0.202^***^ | (0.038) | -0.261^***^ | (0.035) |
| *Interaction effect gender x age* | | | | | | | | |
| 45 years x Female |  |  | 0.271^***^ | (0.053) | 0.221^**^ | (0.079) | 0.318^***^ | (0.072) |
| 60 years x Female |  |  | 0.294^***^ | (0.057) | 0.404^***^ | (0.085) | 0.190^*^ | (0.076) |
| Constant | -28.79^***^ | (0.239) | -28.69^***^ | (0.239) | -28.21^***^ | (0.352) | -29.10^***^ | (0.325) |
| *N* | 35,585 |  | 35,585 |  | 16,879 |  | 18,706 |  |
| *R*^2^ | 0.591 |  | 0.592 |  | 0.586 |  | 0.598 |  |

Data: LINOS-1 & LINO-2 (pooled). Fairness evaluation of earnings regressed on vignette dimensions. Clustered standard errors are reported in parentheses. ^a^ multiplied by 10.

# Online Appendix C


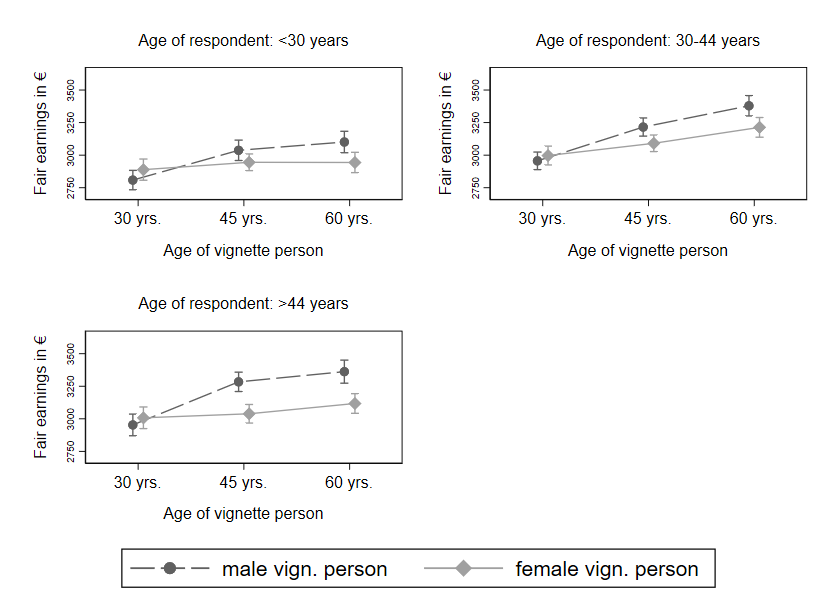


**Figure C1.** Fair earnings for male and female vignette persons by age of vignette person and age of respondents.

*Data: LINOS-1 & LINO-2 (pooled). Fairness evaluation of earnings regressed on vignette dimensions. 95% confidence intervals. The sample was split into three age groups: Respondents below the age of 30 at the time of the interview (n=951), respondents who were between 30 and 44 years old at the time of the interview (n=994), and respondents who were 45 years or older at the time of the interview (n=725).*
